# Automatic Identification of Web-Based Risk Markers for Health Events

**DOI:** 10.2196/jmir.4082

**Published:** 2015-01-27

**Authors:** Elad Yom-Tov, Diana Borsa, Andrew C Hayward, Rachel A McKendry, Ingemar J Cox

**Affiliations:** ^1^Microsoft ResearchHerzeliyaIsrael; ^2^Centre of Computational Statistics and Machine LearningDepartment of Computer ScienceUniversity College LondonLondonUnited Kingdom; ^3^Farr Institute of Health Informatics ResearchUniversity College LondonLondonUnited Kingdom; ^4^London Centre for NanotechnologyUniversity College LondonLondonUnited Kingdom; ^5^Department of Computer ScienceUniversity College LondonLondonUnited Kingdom; ^6^Department of Computer ScienceUniversity of CopenhagenCopenhagenDenmark

**Keywords:** Information retrieval query processing, epidemiology, self-controlled case series, Machine Learning, Web search engines

## Abstract

**Background:**

The escalating cost of global health care is driving the development of new technologies to identify early indicators of an individual’s risk of disease. Traditionally, epidemiologists have identified such risk factors using medical databases and lengthy clinical studies but these are often limited in size and cost and can fail to take full account of diseases where there are social stigmas or to identify transient acute risk factors.

**Objective:**

Here we report that Web search engine queries coupled with information on Wikipedia access patterns can be used to infer health events associated with an individual user and automatically generate Web-based risk markers for some of the common medical conditions worldwide, from cardiovascular disease to sexually transmitted infections and mental health conditions, as well as pregnancy.

**Methods:**

Using anonymized datasets, we present methods to first distinguish individuals likely to have experienced specific health events, and classify them into distinct categories. We then use the self-controlled case series method to find the incidence of health events in risk periods directly following a user’s search for a query category, and compare to the incidence during other periods for the same individuals.

**Results:**

Searches for pet stores were risk markers for allergy. We also identified some possible new risk markers; for example: searching for fast food and theme restaurants was associated with a transient increase in risk of myocardial infarction, suggesting this exposure goes beyond a long-term risk factor but may also act as an acute trigger of myocardial infarction. Dating and adult content websites were risk markers for sexually transmitted infections, such as human immunodeficiency virus (HIV).

**Conclusions:**

Web-based methods provide a powerful, low-cost approach to automatically identify risk factors, and support more timely and personalized public health efforts to bring human and economic benefits.

## Introduction

The identification of risk factors that contribute to the onset of a medical condition plays a crucial role in public health planning, disease prevention, and the promotion of health and wellness. Such factors can be behavioral, such as the link between smoking and lung cancer, or biological and social, and are often interconnected though not necessarily causal. Traditional investigations of associations between individuals’ behaviors and disease rely on analysis of historical medical and public health records (eg, birth and death certificates, hospital admissions), clinical cohort studies, and questionnaires. However, they are often constrained by size, and/or limited information on the full range of diseases, and exposures of interest. Assessing diseases where there are social stigmas is problematic, since they may prevent people from attending clinics, for example, sexually transmitted infections and mental health conditions such as anorexia.

In contrast, anonymized Web search engine query logs facilitate studies with cohort sizes that can include millions of users with many thousands of health events and wide-ranging behavioral information. Every day, millions of people search for information on the Internet and leave electronic traces that can be used to characterize their behaviors and interests. Around 80% of US Internet users seek health information online often before they visit health care professionals [1]. Search engine queries, and user queries in general, have been shown to reflect both the activities of people in the virtual world [2] as well as those in the physical one. For example, Ofran et al [2] found a high correlation between the number of searches for specific types of cancer and their population incidence. Similarly, a high correlation was observed between the number of prescriptions sold for a drug and the number of people who search for it [3]. This has led to a body of work using Internet data to study health, in a burgeoning field also termed “infodemiology” [4] or computational epidemiology [5]. Examples of such work include monitoring of influenza activity [6-9], examining the association between weight and bullying and health [10], exposure to underweight celebrities and the development of eating disorders [11], potential adverse effects of medicines [3], and predicting depression and its appearance [12,13]. Thus, Internet data and especially anonymized search engine query logs provide an unprecedented opportunity to study public health.

The self-controlled case series (SCCS) [14] approach is increasingly used in analysis of large health datasets to investigate the association between a transient exposure and a health event. The method uses only cases (without separate controls), which act as their own controls. SCCS calculates the relative hazard of the health event in the risk period following exposure compared to other time periods. Our work builds on this approach to automatically analyze search engine query logs to identify risk markers for health conditions.

## Methods

### Overview

Our goal was to identify precursor behaviors associated with specific medical conditions. We refer to the proposed solution as the Search Log Analysis for Precursor Behaviors (SLAPB) method, which involves a three-staged process. First, we identified a population of users who were likely to have a medical condition of interest as well as the date this was first acknowledged. Second, we generalized user queries into categories. Finally, we analyzed users' query streams to find likely precursor behaviors. In the following, we describe the three stages.

### Data

We extracted all English language queries submitted to the Bing search engine by users in the United States for the 6-month period starting May 2012. Users agreed to provide their search data as part of the terms of use. Data were anonymized by hashing, before access was granted to the investigators, and aggregated prior to analysis. For each query, we extracted the query text, time and date, a list of pages visited by the user as a result of the query, the zip code of the user (identified according to their IP address), and an anonymized user identifier. A small proportion of users provided information on their year of birth as part of their online profile. No further information, such as gender, were available from these data.

The research was reviewed by the Microsoft Research Institutional Review Board (IRB9672), and was deemed exempt.

### Identification of Populations With Specific Health Conditions

Our first task was to discover a population that has a medical condition of interest, given the queries made by users. We note that we did not seek to diagnose users. We assume that they have already sought medical attention to provide diagnosis and that their queries reflect this. Our goal in this stage was to find a group of users for whom precursor behaviors could be discovered.

We obtained a list of medical terms, which included symptoms, disease names, and drug names, in order to identify medical concerns in each user query. We used the list of 195 medical symptoms and their synonyms from Yom-Tov and Gabrilovich [3]. This is a list obtained from Wikipedia entries of ICD-10 (International Classification of Diseases) symptoms, and expanded through behavioral analysis of Web queries. The list was validated by medical professionals as detailed in Yom-Tov and Gabrilovich [3].

We extracted all the diseases and drug names that appear in Wikipedia through their “DBPedia” entries. Additionally, we augmented each disease and drug through their redirection entries, which serve as synonyms for these entries. A total of 5521 diseases and 5195 drugs were found, together with a total of 26,639 disease name synonyms and 30,409 drug name synonyms. Synonyms were mapped to the relevant disease or drug name.

Using this list, we represented each user by a 10,911-dimensional vector, where each entry is the number of times each symptom, disease, drug, or their synonyms were mentioned in queries made by the user during the data period. Synonyms were counted with the original term.

First, we focused on a subpopulation of users who identified in their queries that they had a specific health condition. Such users queried, for example, “I was diagnosed with HIV [human immunodeficiency syndrome]”. We marked as self-identified users (SIUs), those users who queried for a disease name or medical condition in conjunction with one of the following phrases: “I have”, “I suffer from”, “living with”, and “I was diagnosed with”. We focused on health conditions with at least 25 SIUs, and thus analyzed a population of 18,859 users who self-identified as having one of 92 medical conditions.

We make two very strong assumptions: (1) such users have the health event, and (2) that the onset or diagnosis of the health event occurred at or very near to the time the user made the first query identifying them as an SIU. Of course, neither assumption is valid for all users. However, we believe that the assumptions are, in the majority of cases, valid.

To investigate the validity of our first assumption, that is, that self-identified users experienced the associated health event, we compared the incidence in the United States of 29 diseases as reported in the scientific literature with the corresponding incidence in the SIU group. Table 1 lists the 29 diseases, in rank order, together with their incidence. Table 1 also lists the corresponding incidence and rank in the SIU group. Comparing the relative incidence produces a Spearman rank correlation of rho=.47 (*P*=.005). Though this is a relatively high correlation, we note that it was imperfect for several reasons, including the prominence of that disease in the media, the generality of the term (“cancer” versus “gall bladder cancer”), and sample representativeness. The second assumption was previously made, and validated, in Ofran et al [2].

The 18,859 users labeled as SIU (and having one of 92 events) was not sufficiently large to conduct the subsequent analysis. Thus, in order to identify a larger group of users who are very likely to have experienced a health event, we used the set of SIUs as a training set, and trained a linear support vector machine (SVM) to classify other users. We represented each user as described above.

This is a very high dimensional feature vector, comprising the number of times each user queried for one of the 5521 diseases, 5195 drugs, and 195 symptoms or their synonyms. Thus, we performed an intermediate analysis to determine which of these features were most important for determining the health event associated with each SIU. We constructed a classifier based on these features, and attempted to predict the health event of a test set population. We constructed a linear SVM (using the LIBSVM [15] classifier with default settings) to predict the condition identified by the SIUs, and tested its performance using five-fold cross validation [16] on the 18,859 SIUs.

The results of classifying users into their diseases are shown in Table 2. As this table shows, the most informative features are disease names. The performance of the classifier when using only disease names reaches slightly over 88% correctly classified users. This is in line with the observation that SIUs ask about their condition 6.8 times, on average, compared to 2.15 in the general population of users who asked about specific diseases. Adding features describing drugs and/or symptoms adds little to the classifier’s performance. This is surprising and may be due to the fact that both symptoms and drugs may map to multiple diseases. Future work will investigate how this mapping might be integrated into the classifier, possibly through a hierarchical classification scheme.

**Table 1:** Disease incidence^a^in the United States and in the self-identified user (SIU) population for 29 diseases.

Disease	Percentage of SIUs	Incidence in the United States	Rank of SIU	Rank of incidence in the United States
HIV	6.17E+00	1.53E-04	1	18
Cancer	5.19E+00	5.37E-03	2	3
Diabetes mellitus	4.42E+00	6.13E-03	3	2
Herpes simplex	2.34E+00	2.50E-03	4	4
Arthritis	1.30E+00	4.10E-04	5	11
Atrial fibrillation	1.08E+00	6.45E-02	6	1
Gastroparesis	1.05E+00	2.50E-05	7	28
Heart failure	9.70E-01	1.77E-04	8	15
Schizophrenia	9.44E-01	7.00E-04	9	8
Crohn’s disease	8.38E-01	7.90E-05	10	20
Dementia	7.69E-01	1.51E-03	11	5
Alzheimer's disease	7.53E-01	1.26E-03	12	6
Amyotrophic lateral sclerosis	6.04E-01	1.81E-05	13	29
Parkinson's disease	5.62E-01	1.77E-04	14	15
Breast cancer	5.04E-01	7.51E-04	15	7
Colitis	4.40E-01	8.80E-05	16	19
Asthma	4.19E-01	7.00E-04	17	8
Epilepsy	3.92E-01	4.70E-04	18	10
Lyme disease	3.45E-01	7.00E-05	19	23
Hepatitis C	3.08E-01	5.48E-05	20	25
Spina bifida	2.65E-01	3.49E-04	21	12
Leukemia	2.55E-01	1.69E-04	22	17
Hypothyroidism	2.33E-01	2.86E-04	23	13
Chronic pancreatitis	2.28E-01	4.35E-05	24	27
Celiac disease	2.17E-01	6.50E-05	25	24
Cardiomyopathy	1.96E-01	5.00E-05	26	26
Multiple myeloma	1.59E-01	7.76E-05	27	21
Lymphoma	1.48E-01	2.58E-04	28	14
Brain tumor	1.22E-01	7.54E-05	29	22

^a^Incidence is provided as a fraction of the population.

**Table 2:** Percentage of correctly classified self-identified users (SIUs) given users’ queries for drugs, diseases, symptoms, and their combinations (n=18,859).

Attribute	Correctly classified, n (%)
Drugs	6751 (35.80)
Diseases	16,652 (88.30)
Symptoms	6695 (35.50)
Drugs and diseases	16,671 (88.40)
Drugs and symptoms	6714 (35.60)
Diseases and symptoms	16,675 (88.42)
All three attributes	16,659 (88.33)

An analysis of the most common errors in classifying SIUs given the profile of diseases each user queried for, shows that some of the 12% of errors might not be considered actual errors. Table 3 shows the top five errors of the classifier, which account for 30% of the classifier errors. Many are related to precursor diseases to HIV—CHILD syndrome is an early symptom of acquired immune deficiency syndrome (AIDS), as is HIV. Cancer might refer to Kaposi sarcoma, a tumor often associated with AIDS.

**Table 3:** Five most common errors of the classifier, which predicts SIUs given the disease profile.

Self-identified disease	Predicted disease	Percentage of errors
AIDS	HIV	9.6
AIDS	CHILD syndrome	7.1
AIDS	Pregnancy	6.5
HIV	AIDS	3.6
AIDS	Cancer	3.2

Based on this analysis, we used a much smaller 8-dimensional feature vector (see Table 4) to represent each user. We remind the reader that at this stage our goal was to build a classifier to answer the following question: How likely is the user to be suffering from the health event they most frequently queried for? For example, if a user queried 10 times for “breast cancer” and twice for “flu”, the classifier would give the likelihood that the person is suffering from breast cancer given the disease names “breast cancer” and “flu”, as well as the number of times each was queried. Given this likelihood, we can then determine whether users not in the SIU set have also experienced a health event, and thereby significantly increase the size of the population that has experienced a particular health event. The classifier is trained on the SIU set to distinguish between the 72.2% of cases where the most frequently queried condition is the self-identified condition and the remaining, where this is not the case.

We used five-fold cross validation using the set of self-identified users to test the classifier. Both a linear classifier and a decision tree were tested and both obtained similar performances. Therefore, we report the result of the linear classifier.

The area under the receiver operating characteristic (ROC) curve (AUC) obtained by the classifier using all eight features was 0.851. After running sequential forward feature selection [17], we found that an AUC of 0.855 (not statistically significantly different from the AUC using all the features) could be reached by using only three features, numbered 1, 2, and 5 in Table 4.

Thus, our classifier can accurately predict whether the most frequent disease or medical condition that a user asks about is the one he or she has. However, it may be that the population of SIUs is not representative of the entire patient population. Therefore, we collected from the incidence rates reported in the literature for 29 diseases. Our goal was to tune the classifier such that the relative incidence rate for diseases in the test group would be similar to the relative incidence rates in the general population. By setting a lower threshold of the classifier to decide if a user is suffering from a health condition, larger parts of the user population will be deemed as having a health condition.

**Table 4:** Attributes used to predict whether the most frequently queried disease is the one afflicting the user.

Index	Attribute
1	The number of times queried for the most common diseases.
2	The number of times queried for the second most common diseases.
3	The ratio between the above two.
4	The fraction of users who asked about the most common disease queried by the user.
5	The number of diseases that were asked about more than once.
6	The number of diseases that were asked about more than five times.
7	The number of diseases that were asked about more than 10 times.
8	Indication of queries for drugs related to the most common disease queried by the user: Let *M* be a matrix of co-occurring diseases and drugs, where *Mi,j* is the number of users who asked about disease *i* and also about drug *j*, and let *D* be a matrix of queries for each disease, where *Dij* is whether user *i* asked about drug *j*, we compute an indicator of whether the user asked about drugs related to the most common disease they asked about, by multiplying the row vector of *D* for each user by the matrix *M*.

**Figure 1:**
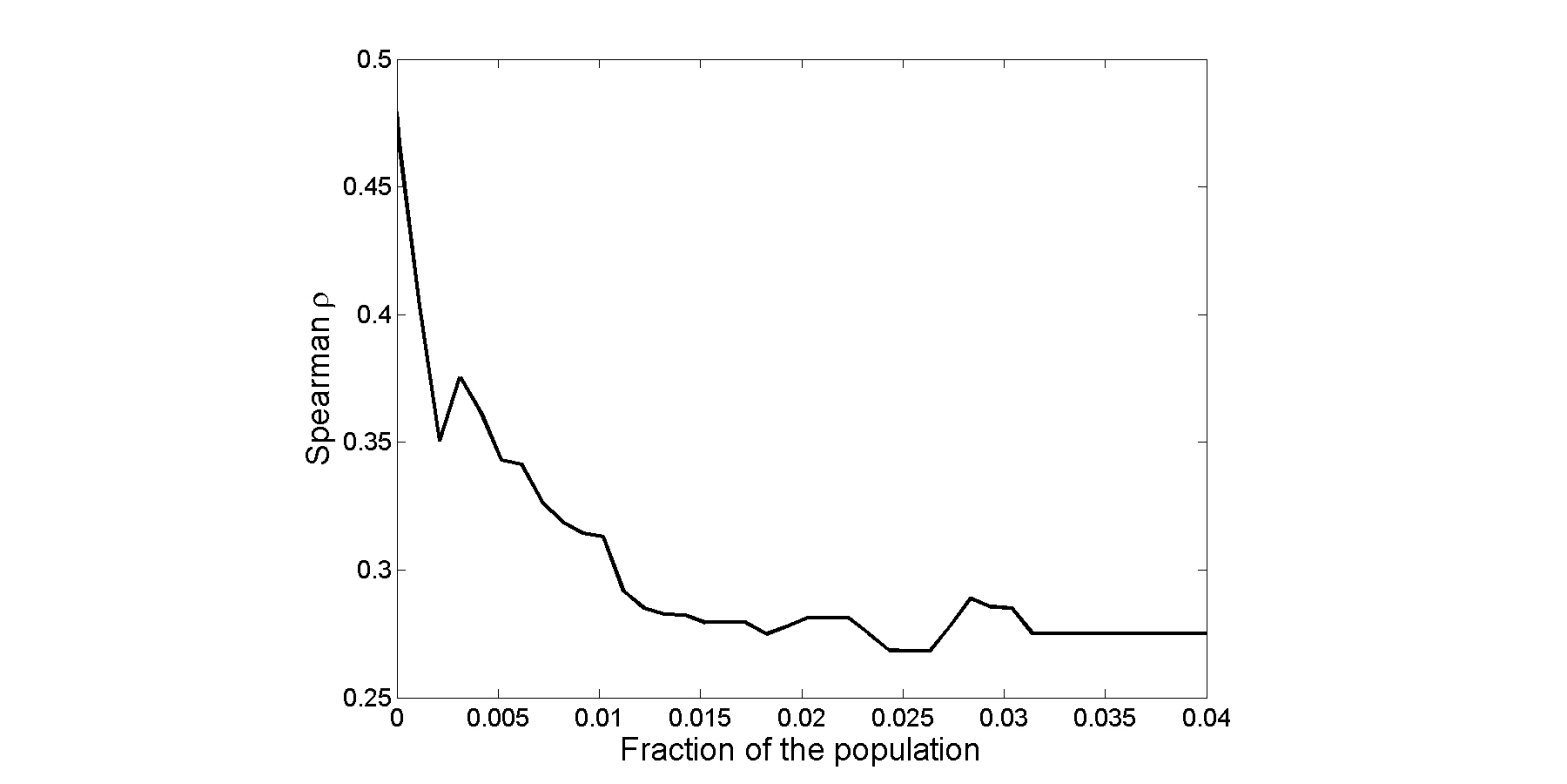
Spearman correlation between known disease incidence (n=29) and the size of the population identified by the classifier, as a function of the classifier threshold.

The Spearman rank correlation between the SIU population for these diseases and the incidence data is rho=.48 (*P*=.0055). Figure 1 shows the dependence of the Spearman correlation on the fraction of the population determined to have a health condition. Here, a threshold of zero denotes the use of only the SIU population and, for any threshold greater than zero, all users for which the classifier gave a score greater than the threshold are identified as having the medical condition they most frequently queried about.

The correlation is strongest for the SIU population. However, a correlation of rho=.32 is achieved for a threshold of around 0.007, with the advantage of having a population of users who are positively classified is 10.6 times greater than the SIU population, thus providing much more data for the next steps of the analysis. Thus, in the paper we use this threshold for identifying populations having specific conditions. We note that the classifier working at this threshold, when applied only to the SIU population, gives a true detection rate of 49.9% and a false alarm rate of 4.5%, that is, 49.9% of the SIU population who queried most often about their self-identified condition are identified as such by the classifier, at the cost of including 4.5% of the SIU population who queried most often about a different condition than their self-identified one.

### Categorization of Web Queries

In this stage of the process we wanted to generalize users’ queries so that broader categories of precursor behaviors might be discerned. For example, suppose users query for beaches to find where to go swimming. The hope is that our algorithms will be able to identify that, for example, swimming is a risk factor and not only that swimming at any specific beach is dangerous. Therefore, we required a way to automatically find a general category, for example, for all cigarette brand names and annotate all queries that describe a cigarette brand name with this category.

To categorize a query, we used query log information in conjunction with Wikipedia entries. We proceeded as follows. First, we found all queries (in the entire dataset) that resulted in users clicking on a specific Wikipedia page. We then found those queries that resulted in the same Wikipedia page being clicked on at least 10 times each month. If more than one Wikipedia page was clicked in response to a query, we chose the one clicked most often. The result of this stage is a list of queries and a corresponding Wikipedia page. For example, the query “pre-diabetes symptoms” is thus associated with the Wikipedia page on pre-diabetes [18].

Finally, each Wikipedia page from the above list is annotated by the Wikipedia categories of that page, as obtained from DBPedia. The above-mentioned query would thus be categorized with the following categories: (1) diabetes, (2) medical conditions related to obesity, and (3) nutrition.

Using this procedure, we categorized a total of 2,375,559 unique queries with one or more of 340,947 categories. In the following, each query made by a user is represented by both its text and by the categories associated with the query, if these are available. Note that not all queries could be annotated because in some cases users didn’t click on a Wikipedia page when they made the query. In those cases, the query is not generalized and only its text is used as a category by itself.

### Discovering Precursor Behaviors: The Self-Controlled Case Series Method

At this point, we have identified users who experienced a health event of interest. We then separately analyzed each health event. For a given health event, we have a corresponding set of users who we have identified as experiencing this event, and, based on their query logs, we have a corresponding set of queries, which we represent by the query text and the associated categories identified in Step 2. For each query text and each category, we then ask whether the specific text/category increases the risk of experiencing the health event. We use the SCCS method to answer this question.

The SCCS method was proposed by Farrington [14] as a method for estimating vaccine safety by measuring the relative incidence of adverse events after a vaccine. As its name suggests, instead of comparing a treated population (for example, one which received a vaccine) against a control population that didn’t receive the treatment of interest, SCCS compares the treatment population against itself, albeit at different times.

In the following, we provide a brief description of SCCS. More details can be found in Madigan et al [19]. Assume N people, denoted by *i*=1,2,…,N, are treated with a given drug. Some of them develop a medical condition, possibly in response to the drug. Each person is observed over a period of *D*_*i*_days. The number of events (eg, medical condition of interest) on a specific day *d* is denoted by *y*_*i,d*_and the day on which person *i* was exposed to the drug by *x*_*i,d*_. If *x*_*i,d*_=1, user *i* was exposed to the drug on day *d*.

SCCS assumes that the medical condition arises according to a non-homogeneous Poisson process, modulated by the drug exposure. Each person *i* has a baseline rate for developing the medical condition, *e* ^*ɵi*^. Exposure to the drug causes a multiplicative effect of *e* ^*β*^on the baseline rate for a period of time, which is referred to as the risk (or incubation) period.

Assume two users are observed over a given time period (D_i_equal to 6 months in this paper). Each is exposed to a drug on a day where *x*_*i,d*_=1, which causes a possible risk for the development of a medical condition on a day when *y*_*i,d*_=1.

**Figure 2:**
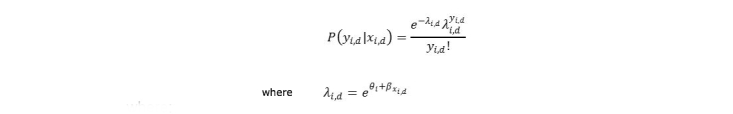
Self-controlled case series (SCCS) density equation.

**Figure 3:**
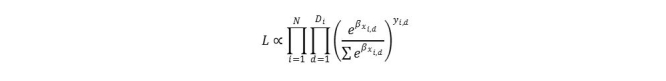
Self-controlled case series (SCCS) maximum likelihood equation.

The number of events observed for person *i* on day *d* has the density as shown in Figure 2. In order to find the parameter of interest, *β*, the log-likelihood over all people is maximized. Thus, we seek to maximize according to Figure 3. We refer to *e* ^*β*^(the parameter we aim to estimate) as the relative incidence or relative hazard of an event [17]. In our work, we used the Nelder-Mead simplex method [20] to maximize *L*.

We applied the SCCS method to our data by searching for precursor events to the development of a specific medical condition of interest. Rather than begin with a specific precursor event, we estimated the relative incidence of all categories and queries (as described in the previous section) available in the data. Thus, for a particular health event, we examine all individuals, *i*, that we identified as experiencing this event. For all individuals, the number of occurrences of the event, *y*_*i,d*_=1, and is only 1 on the day the individual first self-identified or queried about the event. Then, for each category and query associated with the event, we determined the relative hazard, *e* ^*β*^.

One problem that arises in assessing the relative incidence of each category using a relatively limited time span is that transient events may occur that will appear related to the medical condition, when in fact they are related to external factors. For example, news events are known to have a typical spiking behavior with an average length of 3.1 days in search engine queries [21]. If, by chance, the peak of a news event appears close to the time of the medical condition, it may seem to be a precursor behavior.

To reduce this effect, we measured the correlation between the number of times per day that each category appears in the target population (eg, the people who have the medical condition of interest) and the number of times per day it appears in a random sample of users in the entire dataset. We then rejected any category that had a higher correlation than that expected by chance according to the False Discovery Rate (FDR) test [22]. We refer to this step as temporal filtering.

## Results

### Summary

We analyzed seven health conditions, termed “events” shown in Table 5. This table further provides the numbers of users classified as experiencing the event, the number of queries, and corresponding categories. The latter were derived from the Wikipedia categories of pages most strongly associated with each query. For each health event, we identified search categories queried for by at least 1% of the population experiencing the event. We applied the SCCS method to our data by estimating the relative incidence of the health event in the 15 days following each of these search category queries compared to other time periods. Precursor search categories or specific query terms that precede the health event with an FDR of <5% and the corresponding relative hazards are shown in Table 6.

Statistically significant precursor behaviors were identified for each of the seven health events. Many of these behaviors are either known or suspected. In some cases, the method raises plausible new behaviors, which need to be confirmed through traditional epidemiology. In some cases, new online behaviors are identified for which there do not appear to be causal relationships. Of course, this is a manifestation of the limits of such an analysis in which correlation, rather than causation, is used to generate hypotheses.

The following paragraphs discuss the results for each health event. Note that the last column of Table 6 provides the relative hazard, which is defined above.

The most common medical condition identified was pregnancy and as expected searches indicating interest in pregnancy (“fertility” and “pregnancy symptoms”) preceded pregnancy, supporting the validity of our method. The categories “Pregnancy with abortive outcome” and “medical emergencies” may relate to concerns about pregnancy outcomes. Mechanisms for a link with “Teen pregnancy in film and television” are unclear.

The second most common event was allergy. Several preceding search categories associated with allergy are related to household pets (a known allergen for some people) [23]. These include names of pet stores (Petco and PetSmart) and the general category of “pet stores”. The broad food category identified may be indicative of specific foodstuffs that are allergenic.

We investigated two sexually transmitted infections: HIV and herpes simplex (genital herpes simplex is sexually transmitted but oral herpes simplex is not). Two of the preceding search categories associated with both diseases were dating websites and the consumption of adult content (RedTube and Xtube). Studies have found a link between accessing adult sites and risky sexual behavior, leading to an increase in the risk of sexually transmitted infections (STIs) [24]. Heterosexual dating websites (eg, Plenty of Fish) were identified as preceding search categories for herpes, while homosexual dating sites (eg, Adam4Adam) were identified for HIV. Online dating has been suspected to be a risk factor for STIs [25], though evidence is inconclusive. Queries related to real estate were linked to herpes and may be plausibly related if having a new sexual partner prompts individuals to move to a new property. Alternatively, reactivation of oral herpes (cold sores) is known to be related to stress and moving house is a known stressor. The links with “military brats” (a term used to describe the child of a parent or parents serving full-time in the United States Armed Forces) and hip hop music may indicate sexual risk behavior in those associated with these cultures. The link with Walmart may reflect that their pharmacies offer advice on self-management of both oral and genital herpes. Mechanisms for links with popular search engines are unclear.

Myocardial infarction was linked to searches related to restaurants. There is some evidence that the availability of fast-food restaurants partially explains cardiovascular disease prevalence [26]. Our method, however, identifies a transient increase in the risk of myocardial infarction following such searches, suggesting that this exposure goes beyond a long-term risk factor and may also act as an acute trigger of myocardial infarction.

Finally, the correlations with mental health disorders including post-traumatic stress disorder and anorexia found statistically significant precursor behaviors; rape and homelessness queries were preceding search categories for post-traumatic stress disorder and are plausible triggers of this condition. Image search was the strongest preceding search category for eating disorders. Images designed to inspire weight loss, also known as “thinspiration”, are indeed an important part of anorexic behavior [27]. Depression and bipolar disorder were significant preceding search categories previously associated with eating disorders [28]. “English child actors” might have been identified because one of the young English actors in a major movie, which was released several months prior to the data period, is reported to suffer from eating disorders. The link to “Barnes & Noble”, a major Web-based store for books and other media, may indicate content consumed by these users.

**Table 5:** Medical conditions analyzed for precursor behaviors.

Condition	Number of users	Number of queries	Number of categories
Pregnancy	56,062	3,154,273	1263
Allergy	3739	217,395	1455
HIV	1522	80,537	1008
Herpes simplex	709	45,669	1102
Myocardial infarction	701	36,552	1340
Post-traumatic stress disorder	657	36,986	925
Eating disorder	615	37,948	1671

**Table 6:** Precursor search categories and queries associated with the analyzed medical conditions (at FDR rate of 0.05). Queries related to interest in specific people afflicted with the medical condition were manually removed from the list.

Condition	Precursor behaviors	Category or query	Relative hazard
**Pregnancy**			
	Pregnancy symptoms	Query	3.33
	Birth control	Category	2.74
	Fertility	Category	2.58
	Pregnancy with abortive outcome	Category	2.07
	Medical emergencies	Category	1.84
	Teen pregnancy in film and television	Category	1.59
**Allergy**			
	Petco	Query	3.88
	Pet stores	Category	3.34
	Crops originating from the Americas	Category	2.88
	PetSmart	Query	2.07
**Eating disorder**			
	Image search	Category	8.14
	Bipolar spectrum	Category	8.01
	Depression	Category	6.66
	Barnes and Noble (Web-based book store)	Query	4.54
	English child actors	Category	3.85
**Herpes simplex**			
	WorldStarHipHop (multimedia website)	Query	6.12
	Web-based real estate companies	Category	3.50
	Real estate valuation	Category	3.50
	Military brats (children of parents serving full time in the US Armed forces)	Category	2.52
	Plenty of Fish (dating website)	Query	2.34
	Yahoo	Query	2.13
	Zillow (real estate website)	Query	2.06
	Google	Query	2.03
	Walmart	Query	1.94
	Redtube	Query	1.49
**HIV**			
	Xtube	Query	5.50
	Same sex online dating	Category	3.54
	Adam4Adam	Query	3.42
	Video game franchises	Category	3.14
**Myocardial infarction**			
	Fast-food hamburger restaurants	Category	5.28
	Theme restaurants	Category	4.22
**Post-traumatic stress disorder**			
	Homelessness	Query	14.52
	Rape	Query	14.52

### Additional Evidence for the Accuracy of the Proposed Method

#### Geographic and Demographic Variability of the Self-Identified User Population

We used the year of birth provided by a fraction of the Bing users to compute their age, and compared the fraction of users at each (computed) age in the SIU population who reported suffering from arthritis to the distribution of ages in patients with arthritis provided by CDC [29]. The correlation between the two distributions is Spearman rho=.83 (*P*=.06), indicating a good match between the two distributions.

**Figure 4:**
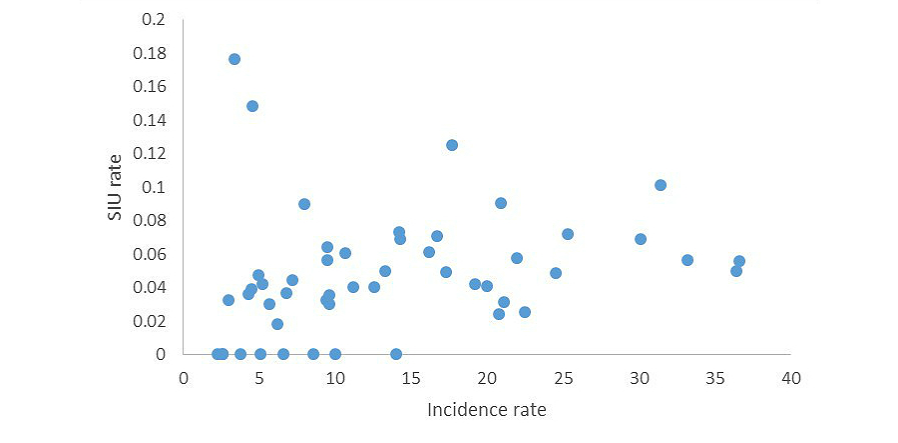
Self-identified user (SIU) rate for users reported having HIV, compared to the HIV incidence rate by state. The correlation between the two variables is Spearman rho=.452 (P=9*10-4).

Similarly, we used the US state in which users resided to find the fraction of SIUs from the population of each state. This information was compared to the prevalence of HIV in each state (as reported by CDC [30]), as shown in Figure 4. The correlation of HIV incidence with SIU fraction is Spearman rho=.452 (*P*=9*10^-4^).

#### Biological Gradient

For many risk factors, greater exposure leads to greater incidence of the effect. For example, exposure to more sexual partners increases the likelihood of contracting a sexually transmitted disease. To validate if such biological gradients exist in the precursors identified in our work (Table 5), we proceeded as follows. For each precursor and condition, we measured the fraction of users identified as having the condition of interest from all users who queried for the precursor, stratified by the number of times they searched for the precursor.

We then categorized these precursors according to whether there was a positive correlation (rho≥.5) between the fraction of users identified with the condition and the number of searches for the precursor, negative (rho<.5), or undetermined. Accordingly, 64% of precursors had a positive correlation and 22% a negative one. Thus, the majority of precursors show a biological gradient of an elevated likelihood for a medical condition associated with an increased interest in the precursors.

## Discussion

### Principal Findings

The experimental results identified a number of known risks associated with specific health events, providing support for the proposed method. New potential risks factors were also identified that could form the basis for traditional epidemiological investigation and verification. Of course, the risk factors we identified are correlational and not necessarily causal. In fact, with very few exceptions, no online behavior will ever be directly causal of a health event.

Our approach minimizes the role of chance, bias, and confounding. The large numbers of search engine users reduces the role of chance. The SCCS approach, whereby people with the health event act as their own controls, largely eliminates selection bias (systematic differences in how cases and controls or exposed and unexposed individuals are selected for the study). Recall bias is minimized as people’s Internet searches are recorded in real time and likely relate to their current concerns. It is also reassuring that demographic and geographic evidence correlated well with external data (supplementary materials). Similarly, the searches related to precursor behaviors may not reflect genuine behavior. A major advantage of the SCCS is that confounding by factors that do not vary significantly over the study period are implicitly controlled for. Nevertheless, some of the associations may be due to confounders that are temporally related to the identified search categories. The fact that the method identifies search categories preceding the health event helps to clarify the direction of association, but it is likely that the method may not always accurately identify the timing of the events/behaviors of interest. Most of the associations identified meet many of the Bradford Hill criteria [31] for assessing causality including temporality, strength of response, plausibility, and coherence and consistency with other research.

### Conclusions

To conclude, while we recognize that the wider issues of data privacy and the societal acceptability of Web-based studies for health need to be considered, the method is an important scientific breakthrough to providing a faster and more economic means of automatically generating risk factors linking behaviors with the onset of major health conditions. The approach could be adapted to testing of pre-specified hypotheses, over short- and long-term studies. A better understanding of Web-based health-seeking behaviors could also help to direct disease prevention efforts to high risk in the physical or virtual world.

## Abbreviations

AIDS: acquired immune deficiency syndrome
AUC: area under the curve
FDR: false discovery rate
HIV: human immunodeficiency syndrome
ICD-10: International Classification of Diseases, Tenth Revision
ROC: receiver operating characteristic
SCCS: self-controlled case series
SIU: self-identified user
SLAPB: search log analysis for precursor behaviors
STI: sexually transmitted infection
SVM: support vector machine
